# Absence of Fibrin in the Blood

**Published:** 1853-05

**Authors:** 


*Absence of Fibrin in the Blood.*—A singular case of *purpura hæmorrhagica* lately occurred in the wards of M. Hérard, of La Pitiè. The patient was 35 years of age, a gilder by profession. His general health was good; his constitution was strong; his temperament sanguine; he had recovered a fortnight since from an erysipelas of the face, which had exhibited nothing unusual in its progress or symptoms. On the 19th August, after a cold bath, he had a chill, followed by fever, headache, and violent pain in the loins. The 20th and 21st the disease augmented: the patient had nausea and vomiting. The 22d he went on foot to the hospital La Pitiè, where he was admitted. The interne of the ward, M. Labric, found him in the evening after his entrance with intense fever, headache, loss of appetite, a white tongue, and a pain in the loins of such severity that it extorted cries of anguish from the patient, and absorbed almost all of the other symptoms. For the rest, there was no watering of the eyes, nor coryza; no cough, no sore throat, no diarrhea. Here and there upon the face and limbs was an eruption, which gave rise to the suspicion of small pox, but the patient, besides having been vaccinated, asserted that these eruptions had existed for a long time.

The 24th the renal distress and headache were undiminished; the skin was hot, the pulse was full at 108. The conjunctivæ were ecchymosed; a great number of petechiæ covered the lower limbs and abdomen; they were confluent at the hypogaster, sparsely scattered over the breast and arms. Upon the legs, several spots of a bluish tinge denoted profound extravasations The gums were healthy; the patient has spit blood, and declares that his urine is bloody. Auscultation discovered diffused sub-crepitant râles. No appreciable alteration upon percussion. (Lemonade; venesection, ℥vj.; diet.) Immediate relief was experienced after the bleeding. Subsequently, several hours after, the patient complained all at once of a sensation of suffocation, and suddenly died.

*Examination of the blood drawn from the vein.*—The blood did not separate into clot and serum; it preserved, for 24 hours, a dark color, and a most remarkable state of fluidity. There was no trace of the buffy coat. Dr. Becquerel, who made the quantitative and qualitative analysis, arrived at the following altogether unexpected results:

*Fibrin.*—After beating the liquid for quarter of an hour with a willow twig, it was impossible to discover the least atom of fibrin.

*Globules.*—Same impossibility of separating the globules from the serum.

*Density.*—The density of the altered blood was 1053.56. In 1000 parts of blood, evaporation showed the quantity of water to be 803.84; the residue being icinerated, the solid constituents were 196.56.—*Bulletin de l'Académie. Va. Med. and Surg. Jour.*

